# Bis[4-(dimethyl­amino)pyridinium] tetra­bromidocobaltate(II)

**DOI:** 10.1107/S1600536809024398

**Published:** 2009-07-01

**Authors:** Kong Mun Lo, Seik Weng Ng

**Affiliations:** a Department of Chemistry, University of Malaya, 50603 Kuala Lumpur, Malaysia

## Abstract

The metal atom in the title salt, (C_7_H_11_N_2_)_2_[CoBr_4_], shows a slightly distorted tetra­hedral coordination. The cation forms an N—H⋯Br hydrogen bond to one of the two Br atoms. The Co^II^ atom lies on a special position of 2 site symmetry.

## Related literature

For bis[4-(dimethylamino)pyridinium] tetrabromido­cadmate(II) monohydrate, see: Lo & Ng (2009 ▶).
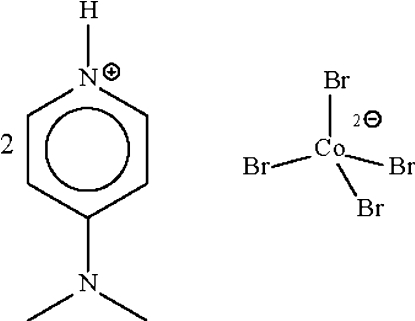


## Experimental

### 

#### Crystal data

(C_7_H_11_N_2_)_2_[CoBr_4_]
*M* *_r_* = 624.93
Monoclinic, 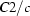

*a* = 10.4020 (2) Å
*b* = 12.1601 (2) Å
*c* = 16.9167 (2) Å
β = 104.270 (1)°
*V* = 2073.76 (6) Å^3^
*Z* = 4
Mo *K*α radiation
μ = 8.54 mm^−1^
*T* = 140 K
0.40 × 0.35 × 0.30 mm

#### Data collection

Bruker SMART APEX diffractometer
Absorption correction: multi-scan (*SADABS*; Sheldrick, 1996 ▶) *T*_min_ = 0.131, *T*_max_ = 0.184 (expected range = 0.055–0.077)
8405 measured reflections
2386 independent reflections
2228 reflections with *I* > 2σ(*I*)
*R*_int_ = 0.024

#### Refinement

*R*[*F* ^2^ > 2σ(*F* ^2^)] = 0.021
*wR*(*F* ^2^) = 0.054
*S* = 1.06
2386 reflections
111 parameters
1 restraint
H atoms treated by a mixture of independent and constrained refinement
Δρ_max_ = 0.49 e Å^−3^
Δρ_min_ = −0.75 e Å^−3^

### 

Data collection: *APEX2* (Bruker, 2008 ▶); cell refinement: *SAINT* (Bruker, 2008 ▶); data reduction: *SAINT*; program(s) used to solve structure: *SHELXS97* (Sheldrick, 2008 ▶); program(s) used to refine structure: *SHELXL97* (Sheldrick, 2008 ▶); molecular graphics: *X-SEED* (Barbour, 2001 ▶); software used to prepare material for publication: *publCIF* (Westrip, 2009 ▶).

## Supplementary Material

Crystal structure: contains datablocks global, I. DOI: 10.1107/S1600536809024398/bt2980sup1.cif

Structure factors: contains datablocks I. DOI: 10.1107/S1600536809024398/bt2980Isup2.hkl

Additional supplementary materials:  crystallographic information; 3D view; checkCIF report

## Figures and Tables

**Table 1:** Hydrogen-bond geometry (Å, °)

*D*—H⋯*A*	*D*—H	H⋯*A*	*D*⋯*A*	*D*—H⋯*A*
N1—H1⋯Br1	0.87 (1)	2.71 (2)	3.454 (2)	144 (3)

**Fig. 1.**
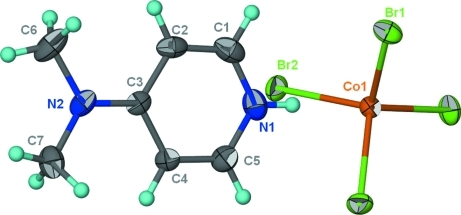
Anisotropic displacement ellipsoid plot (Barbour, 2001) of the title compound at the 70% probability level. H atoms are drawn as spheres of arbitrary radius.

## supplementary crystallographic information

### Experimental

Cobalt nitrate hexahydrate (0.89 g, 3 mmol) dissolved in a minimum volume of water was mixed with 4-dimethylaminopyridinium hydrobromide perbromide (1.1 g, 3 mmol) dissolved in 50 ml ethanol. The mixture was heated for 1 hour. The red solution slowly turned to blue solution. This was set aside for the growth of crystals.

### Refinement

Hydrogen atoms were placed at calculated positions (C–H 0.95–0.98 Å) and were treated as riding on their parent atoms, with *U*(H) set to 1.2*U*~eq~(C). The amino H-atom was refined with a distance restraint of 0.84±0.01 Å.

### Crystal data

**Table:**

(C_7_H_11_N_2_)_2_[CoBr_4_]	*F*(000) = 1204
*M**_r_* = 624.93	*D*_x_ = 2.002 Mg m^−^^3^
Monoclinic, *C*2/*c*	Mo *K*α radiation, λ = 0.71073 Å
Hall symbol: -C 2yc	Cell parameters from 6526 reflections
*a* = 10.4020 (2) Å	θ = 2.5–28.4°
*b* = 12.1601 (2) Å	µ = 8.54 mm^−^^1^
*c* = 16.9167 (2) Å	*T* = 140 K
β = 104.270 (1)°	Block, blue
*V* = 2073.76 (6) Å^3^	0.40 × 0.35 × 0.30 mm
*Z* = 4	

### Data collection

**Table:**

Bruker SMART APEX diffractometer	2386 independent reflections
Radiation source: fine-focus sealed tube	2228 reflections with *I* > 2σ(*I*)
graphite	*R*_int_ = 0.024
ω scans	θ_max_ = 27.5°, θ_min_ = 2.5°
Absorption correction: multi-scan (*SADABS*; Sheldrick, 1996)	*h* = −11→13
*T*_min_ = 0.131, *T*_max_ = 0.184	*k* = −15→15
8405 measured reflections	*l* = −21→21

### Refinement

**Table:**

Refinement on *F*^2^	Primary atom site location: structure-invariant direct methods
Least-squares matrix: full	Secondary atom site location: difference Fourier map
*R*[*F*^2^ > 2σ(*F*^2^)] = 0.021	Hydrogen site location: inferred from neighbouring sites
*wR*(*F*^2^) = 0.054	H atoms treated by a mixture of independent and constrained refinement
*S* = 1.06	*w* = 1/[σ^2^(*F*_o_^2^) + (0.0282*P*)^2^ + 3.2278*P*] where *P* = (*F*_o_^2^ + 2*F*_c_^2^)/3
2386 reflections	(Δ/σ)_max_ = 0.001
111 parameters	Δρ_max_ = 0.49 e Å^−^^3^
1 restraint	Δρ_min_ = −0.75 e Å^−^^3^

### Special details

**Table:**

Geometry. All esds (except the esd in the dihedral angle between two l.s. planes) are estimated using the full covariance matrix. The cell esds are taken into account individually in the estimation of esds in distances, angles and torsion angles; correlations between esds in cell parameters are only used when they are defined by crystal symmetry. An approximate (isotropic) treatment of cell esds is used for estimating esds involving l.s. planes.

### Fractional atomic coordinates and isotropic or equivalent isotropic displacement parameters (Å^2^)

**Table:**

	*x*	*y*	*z*	*U*_iso_*/*U*_eq_	
Br1	0.66045 (2)	0.777934 (17)	0.345907 (13)	0.02380 (7)	
Br2	0.36829 (2)	0.555780 (17)	0.314578 (12)	0.02431 (7)	
Co1	0.5000	0.67093 (3)	0.2500	0.01636 (9)	
N1	0.7776 (2)	0.51482 (16)	0.39578 (13)	0.0268 (4)	
H1	0.746 (3)	0.5670 (19)	0.3610 (16)	0.044 (9)*	
N2	0.94584 (19)	0.27718 (16)	0.56095 (11)	0.0238 (4)	
C1	0.8017 (2)	0.53686 (18)	0.47578 (15)	0.0267 (5)	
H1A	0.7809	0.6076	0.4929	0.032*	
C2	0.8555 (2)	0.46012 (18)	0.53283 (14)	0.0235 (4)	
H2	0.8709	0.4770	0.5892	0.028*	
C3	0.88849 (19)	0.35447 (17)	0.50763 (12)	0.0183 (4)	
C4	0.8593 (2)	0.33468 (17)	0.42225 (12)	0.0194 (4)	
H4	0.8782	0.2650	0.4024	0.023*	
C5	0.8047 (2)	0.41504 (19)	0.36914 (13)	0.0243 (4)	
H5	0.7851	0.4009	0.3122	0.029*	
C6	0.9690 (3)	0.2937 (3)	0.64888 (14)	0.0364 (6)	
H6A	0.8871	0.3194	0.6616	0.055*	
H6B	1.0390	0.3488	0.6668	0.055*	
H6C	0.9966	0.2241	0.6772	0.055*	
C7	0.9865 (2)	0.17184 (19)	0.53287 (15)	0.0289 (5)	
H7A	1.0364	0.1850	0.4916	0.043*	
H7B	0.9076	0.1277	0.5090	0.043*	
H7C	1.0426	0.1323	0.5792	0.043*	

### Atomic displacement parameters (Å^2^)

**Table:**

	*U*^11^	*U*^22^	*U*^33^	*U*^12^	*U*^13^	*U*^23^
Br1	0.01960 (12)	0.02113 (11)	0.02904 (12)	−0.00386 (8)	0.00292 (9)	−0.00728 (8)
Br2	0.03047 (13)	0.02293 (12)	0.02059 (11)	−0.00884 (8)	0.00829 (9)	0.00109 (7)
Co1	0.01504 (19)	0.01626 (17)	0.01751 (18)	0.000	0.00345 (14)	0.000
N1	0.0223 (10)	0.0229 (9)	0.0346 (10)	0.0040 (7)	0.0060 (8)	0.0059 (8)
N2	0.0206 (9)	0.0335 (10)	0.0166 (8)	0.0013 (7)	0.0029 (7)	0.0029 (7)
C1	0.0198 (11)	0.0233 (10)	0.0391 (13)	−0.0010 (8)	0.0110 (9)	−0.0074 (9)
C2	0.0178 (10)	0.0300 (11)	0.0245 (10)	−0.0046 (8)	0.0085 (8)	−0.0094 (8)
C3	0.0112 (9)	0.0249 (10)	0.0190 (9)	−0.0028 (7)	0.0042 (7)	−0.0007 (8)
C4	0.0170 (10)	0.0218 (9)	0.0191 (9)	0.0013 (8)	0.0040 (7)	−0.0012 (7)
C5	0.0214 (10)	0.0290 (11)	0.0212 (10)	0.0004 (9)	0.0030 (8)	0.0005 (8)
C6	0.0301 (13)	0.0608 (17)	0.0166 (10)	−0.0005 (12)	0.0027 (9)	0.0048 (10)
C7	0.0251 (12)	0.0268 (11)	0.0320 (12)	0.0034 (9)	0.0019 (9)	0.0071 (9)

### Geometric parameters (Å, °)

**Table:**

Br1—Co1	2.4033 (3)	C2—C3	1.422 (3)
Br2—Co1	2.4019 (3)	C2—H2	0.9500
Co1—Br2^i^	2.4019 (3)	C3—C4	1.421 (3)
Co1—Br1^i^	2.4033 (3)	C4—C5	1.354 (3)
N1—C1	1.341 (3)	C4—H4	0.9500
N1—C5	1.348 (3)	C5—H5	0.9500
N1—H1	0.872 (10)	C6—H6A	0.9800
N2—C3	1.337 (3)	C6—H6B	0.9800
N2—C6	1.461 (3)	C6—H6C	0.9800
N2—C7	1.464 (3)	C7—H7A	0.9800
C1—C2	1.360 (3)	C7—H7B	0.9800
C1—H1A	0.9500	C7—H7C	0.9800
			
Br2—Co1—Br2^i^	108.678 (18)	C4—C3—C2	116.79 (19)
Br2—Co1—Br1	112.808 (7)	C5—C4—C3	120.1 (2)
Br2^i^—Co1—Br1	104.098 (8)	C5—C4—H4	119.9
Br2—Co1—Br1^i^	104.099 (8)	C3—C4—H4	119.9
Br2^i^—Co1—Br1^i^	112.808 (7)	N1—C5—C4	121.0 (2)
Br1—Co1—Br1^i^	114.444 (18)	N1—C5—H5	119.5
C1—N1—C5	121.0 (2)	C4—C5—H5	119.5
C1—N1—H1	119 (2)	N2—C6—H6A	109.5
C5—N1—H1	120 (2)	N2—C6—H6B	109.5
C3—N2—C6	121.6 (2)	H6A—C6—H6B	109.5
C3—N2—C7	120.84 (18)	N2—C6—H6C	109.5
C6—N2—C7	117.52 (19)	H6A—C6—H6C	109.5
N1—C1—C2	121.4 (2)	H6B—C6—H6C	109.5
N1—C1—H1A	119.3	N2—C7—H7A	109.5
C2—C1—H1A	119.3	N2—C7—H7B	109.5
C1—C2—C3	119.6 (2)	H7A—C7—H7B	109.5
C1—C2—H2	120.2	N2—C7—H7C	109.5
C3—C2—H2	120.2	H7A—C7—H7C	109.5
N2—C3—C4	120.97 (19)	H7B—C7—H7C	109.5
N2—C3—C2	122.24 (19)		
			
C5—N1—C1—C2	0.7 (3)	C1—C2—C3—N2	177.6 (2)
N1—C1—C2—C3	0.7 (3)	C1—C2—C3—C4	−1.6 (3)
C6—N2—C3—C4	−176.2 (2)	N2—C3—C4—C5	−178.1 (2)
C7—N2—C3—C4	3.1 (3)	C2—C3—C4—C5	1.0 (3)
C6—N2—C3—C2	4.7 (3)	C1—N1—C5—C4	−1.2 (3)
C7—N2—C3—C2	−176.0 (2)	C3—C4—C5—N1	0.3 (3)

Symmetry codes: (i) −*x*+1, *y*, −*z*+1/2.

### Hydrogen-bond geometry (Å, °)

**Table:**

*D*—H···*A*	*D*—H	H···*A*	*D*···*A*	*D*—H···*A*
N1—H1···Br1	0.87 (1)	2.71 (2)	3.454 (2)	144 (3)
